# Amplify antimicrobial photo dynamic therapy efficacy with poly-beta-amino esters (PBAEs)

**DOI:** 10.1038/s41598-021-86773-3

**Published:** 2021-03-31

**Authors:** Stefano Perni, Emily C. Preedy, Polina Prokopovich

**Affiliations:** Cardiff School of Pharmacy and Pharmaceutical Sciences, Cardiff University, Redwood Building, King Edward VII Avenue, Cardiff, CF10 3NB UK

**Keywords:** Bacterial infection, Drug delivery

## Abstract

Light-activated antimicrobial agents (photosensitisers) are promising alternatives to antibiotics for the treatment of skin infections and wounds through antimicrobial photo dynamic therapy (aPDT); utilisation of this technique is still restricted by general low efficacy requiring long exposure time (in the order of tens of minutes) that make the treatment very resource intensive. We report for the first time the possibility of harvesting the cell penetrating properties of poly-beta-amino esters (PBAEs) in combination with toluidine blue O (TBO) to shorten aPDT exposure time. Candidates capable of inactivation rates 30 times quicker than pure TBO were discovered and further improvements through PBAE backbone optimisation could be foreseen. Efficacy of the complexes was PBAE-dependent on a combination of TBO uptake and a newly discovered and unexpected role of PBAEs on reactive species production. Chemometric approach of partial least square regression was employed to assess the critical PBAE properties involved in this newly observed phenomenon in order to elicit a possible mechanism. The superior antimicrobial performance of this new approach benefits from the use of well established, low-cost and safe dye (TBO) coupled with inexpensive, widely tested and biodegradable polymers also known to be safe. Moreover, no adverse cytotoxic effects of the PBAEs adjuvated TBO delivery have been observed on a skin cells in vitro model demonstrating the safety profile of this new technology.

## Introduction

Skin and soft-tissue infections (SSTIs) are some of the most common infections observed in ambulatory practice and hospitals; to further concern, their rate has increased in the last decades^1^. SSTI symptoms range from mild conditions, such as pyoderma, to serious life-threatening such as necrotizing fasciitis; besides the affected area (i.e. hands or legs) may lose functionality depending on the severity of the infection^2^. Clearly SSTI are simultaneously a threat to life and quality of life to patients and an economic burden to health care providers^3,4^; i.e. it has been estimated that every MRSA infection costs an extra 9000 £ to the NHS^5^ while in the USA the total spent on the treatment of SSTIs tripled between 2000 and 2012, from $4.4 to $13.8 billion^6^.

Antibiotics are the first line approach to treat SSTI, however their efficacy is rapidly decreasing in consequence of the growth of antibiotic resistance among pathogens. New antibiotics, such as oritavancin or delafloxacin, to treat SSTI, have emerged^7,8^; nonetheless it is expected that antibiotic resistance will outpace the development of new drug thus different antimicrobial treatment is urgently needed.

Photodynamic therapy is a local, repeatable non-invasive technique which might be an effective alternative to antibiotics for the treatment of local infections^9^, periodontitis^10^ and diabetic foot ulcer^11,12^. The mechanism of action of PDT derives from a light-induced excitation of a photosensitiser (PS) to singlet state and its conversion to triplet state, whose reactivity results in the generation of radicals and ROS^13,14^. These cell death inducing capabilities of ROS have found medical applications in cancer^15^ and infections (antimicrobial-PDT or aPDT) treatment^14^.

aPDT feasibility depends on the higher resistance of mammalian cells than pathogens to the oxidation induced by light activated compounds^16,17^ and by biologically compatible light activated compounds PS^13,14^. Lethality mechanism of aPDT is multi-targets; hence it is not prone to induce further resistance^14,18–20^. A further benefit of aPDT is the possibility of inactivating not only viable cells but also virulence factors released by pathogens that can cause tissue damage after bacterial death^21^. This possibility makes aPDT stand alone in the variety of antimicrobial techniques that are capable of only interacting with cells leaving virulence factors untouched. Clinical trials have also investigated aPDT for the treatment of skin infections^22–25^.

Despite the numerous mentioned benefits of aPDT, the necessary exposure time (in the order of 10–15 min)^26^ is currently the main obstacle to its application in hospital settings as this limits the number of patients that can be treated per beds available. The challenge of reducing the irradiation time necessary to achieve aPDT satisfactory results has been addressed by developing delivery systems that augment PS uptake^27,28^ or designing novel PS that either exhibit selective affinity to bacterial cells over other tissue cells or are more potent ROS producers^11,29–31^. Despite the positive results, such advances are not yet sufficient in terms of efficacy or economically viable; consequently, new approaches are required in order to tackle the antibiotic resistance threat in a cost-effective way that maximises the return of health providers scarce resources.

**Figure 1:**
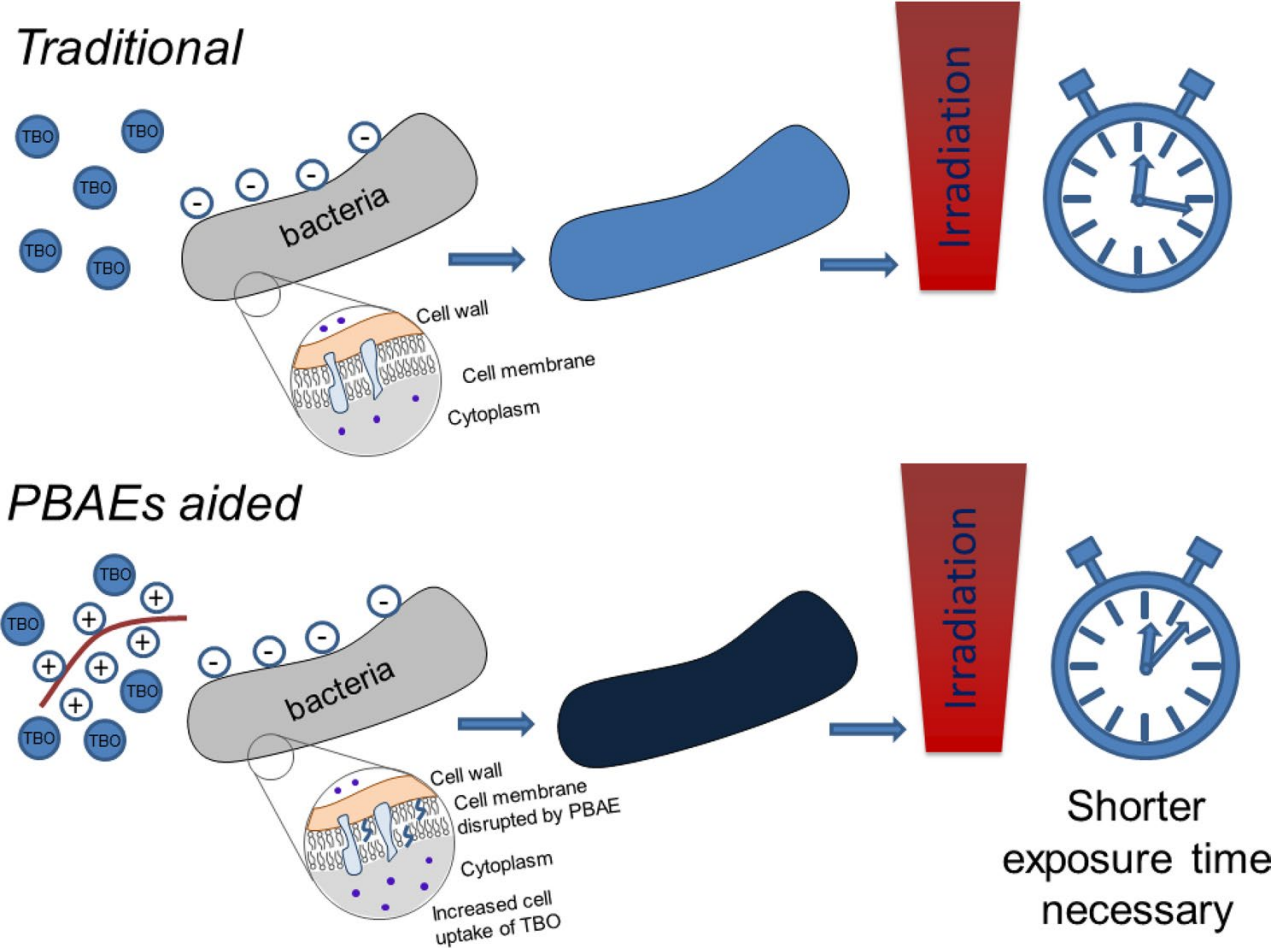
Schematic representation of the mechanism underpinning the enhancement of aPDT efficacy due to PBAE.

Poly-beta-amino esters (PBAEs) were first described as biodegradable and biocompatible polymers with cell membrane penetrating characteristics that could be employed as gene transfer vectors in light of their positive charges and ability to form complexes with DNA molecules^32^. We hypothesised that PBAEs capabilities to actively deliver molecules inside cells could be exploited as adjuvant to microbial cell uptake of toluidine blue O (TBO) and demonstrated for the first time that PBAE-TBO complexes can have higher antimicrobial activity after laser exposure than the comparable amount of free dye on various pathogens representative of SSTI (Fig. 1). TBO was chosen in light of being one of the few light activated compounds already approved for clinical use^14^. We then investigated a library of PBAEs to judge whether optimisation was feasible and identified candidates capable of increasing inactivation rates to > 30 times than those of pure TBO. This has the potential of providing the long-awaited break-through that could have clinical relevance in the treatment of SSTI. We also ascertained key PBAEs features that would allow further targeted development through data analytics algorithms. Finally, the effect on skin cells (keratinocytes and fibroblasts) was assessed in vitro to supplement the antimicrobial efficacy data of the technology with clear safety results.

## Materials and methods

### Synthesis of PBAEs

**Figure 2:**
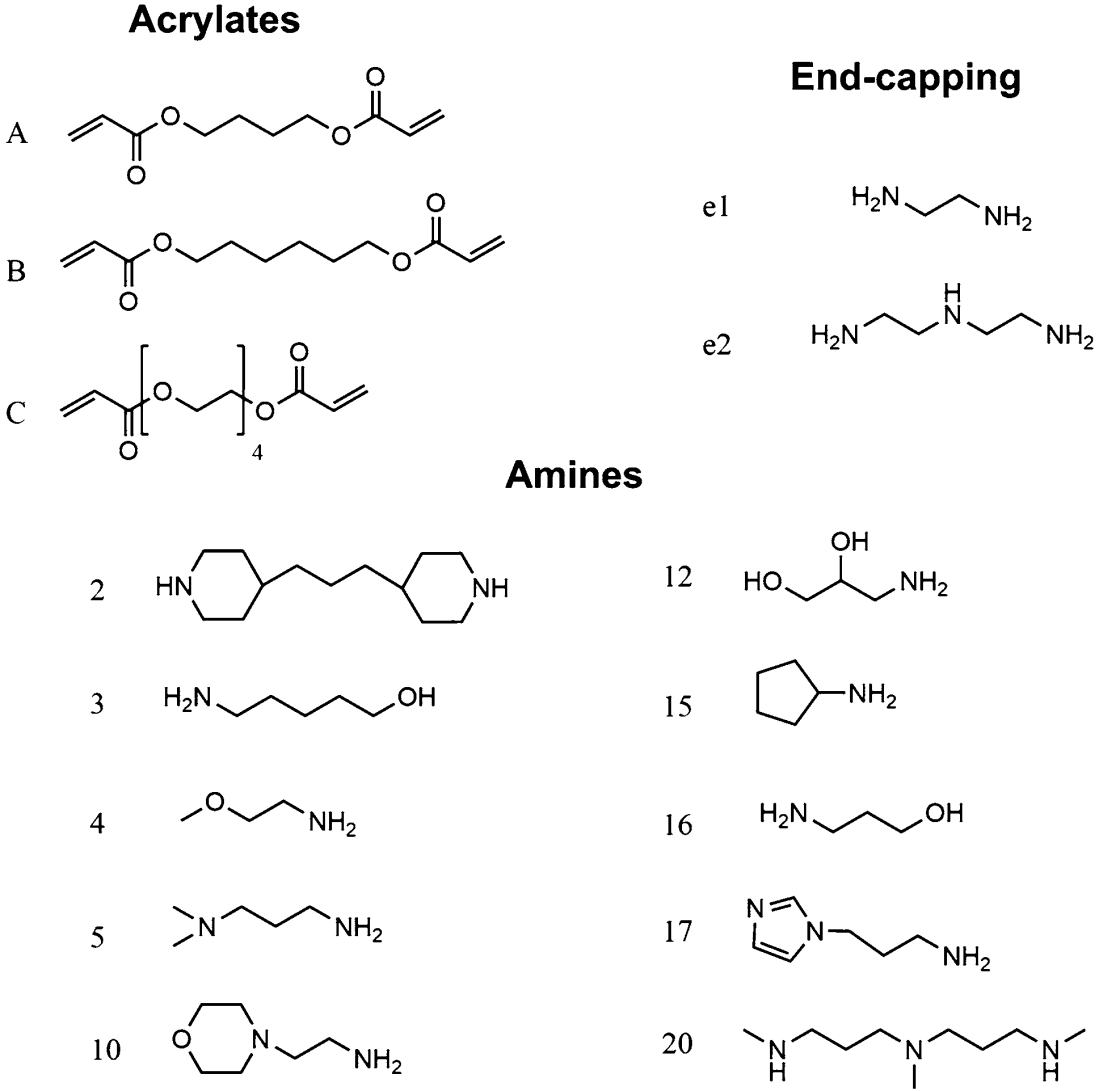
Structure and coding of constituents of PBAEs library used.

**Figure 3:**
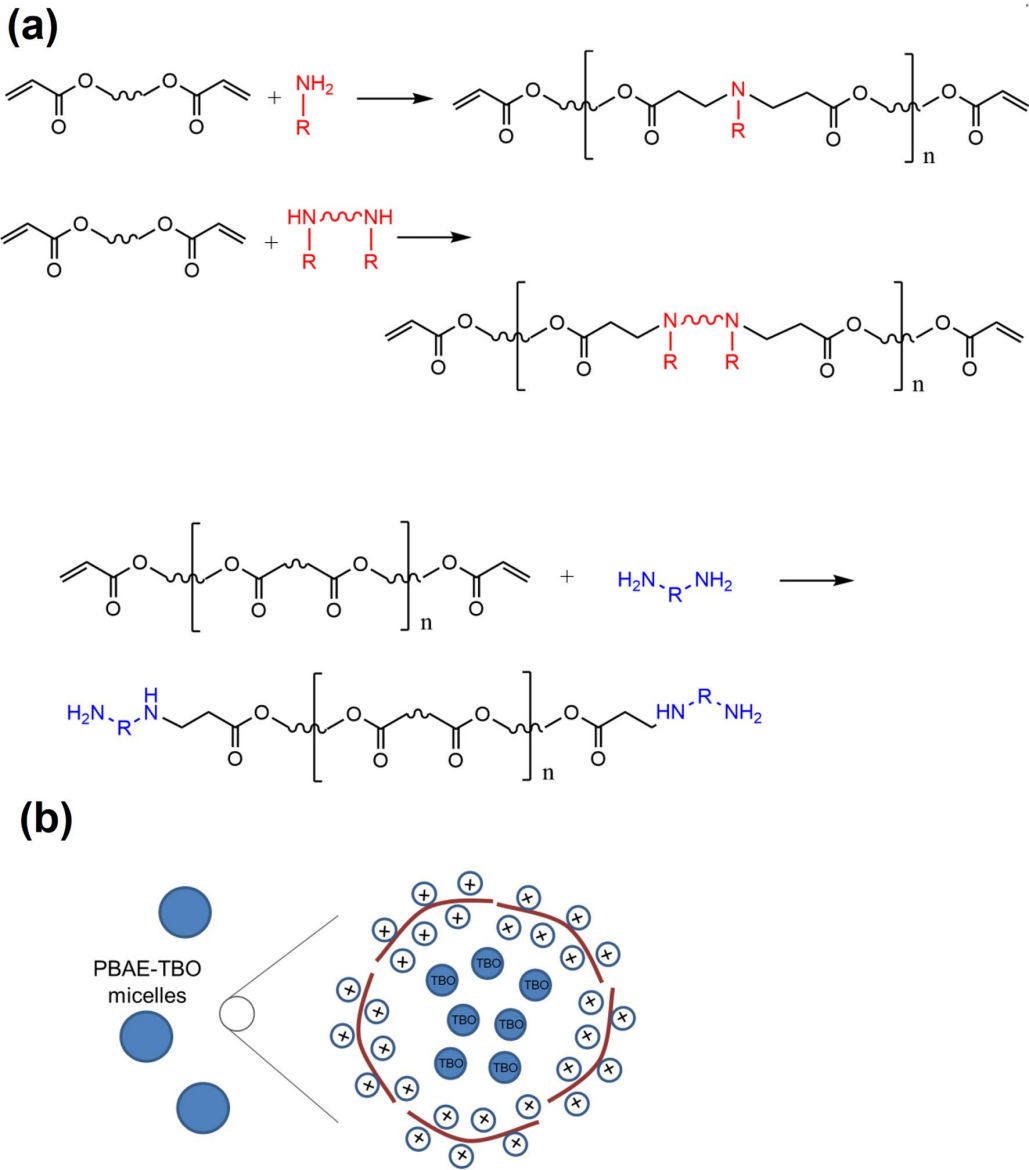
(**a**) PBAE structure and synthesis (**b**) PBAE-TBO micelles.

Acrylate-terminated poly(β-amino ester)s were synthesized by mixing 1,4-butandiole diacrylate, 1,6-hexanediol diacrylate or tetra(ethylene glycol) diacrylate with a range of amine monomers (Fig. 2) in dichloro-methane (DCM) with a acrylate excess^33,34^. The polymerisation, a Michael addition reaction (Fig. 3), was performed under stirring at 50 °C for 48 h. PBAEs were precipitated through pouring the reaction mixture in about 10 times the volume of diethyl-ether under vigorous mixing; the solvent was then removed under vacuum.

Acrylate-terminated polymers of were dissolved in DCM at a concentration of 31.13% w/w. A range of end capping agents (Fig. 2) were then added^33,34^. End-capped PBAEs were recovered through precipitation in diethyl-ether under vigorous mixing, the unreacted amines were removed centrifuging the suspension of PBAE in diethyl-ether/DCM for 2 min at 1155*g*. The supernatant was removed and the PBAEs washed twice with diethyl-ether. The end-capped PBAEs were then dried under vacuum.

The PBAEs and derivatives obtained were coded through the constituents of the backbone using a capital letter to indicate the acrylate (e.g. A being 1,4-butanediol acrylate) and a number to indicate the amine as shown in Fig. 2; the further end-capping is describes by a number preceded by the letter e. For example, A2-e1 is the PBAE obtained from 1,4-butanediol diacrylate and 4,4′-Trimethylenedipiperidine then capped with ethylene-diamine.

### PBAE characterization

Gel permeation chromatography (GPC) was employed to determine the molecular weight of each PBAE in the library. Samples were injected in a Shimadzu-LC-20Ai system equipped with a Superdex 75 10/300 GL column; the mobile phase was 100% sodium acetate buffer pH = 5 at a constant flow of 1 mL/min. Number-averaged (*Mn*) and weight-averaged molecular weight (*Mw*) were determined against PEG standards. Molar masses were only apparent values due to the use of PEG standards and possible different hydrodynamic volumes of PEG standards and poly(beta-amino esters) of identical molar mass^34^.

### Preparation of TBO–PBAEs complex

10 mg of TBO were dissolved in DCM (25 mL) along with 100 mg of end-capped polymer; the solutions were immediately covered with aluminium foil and stirred for 24 h at room temperature. The PBAEs-TBO derivatives were recovered through precipitation in diethyl-ether under vigorous mixing, the unreacted molecules were removed centrifuging the suspension of PBAE in diethyl-ether/DCM for 2 min at 1155*g*. The supernatant was removed and the PBAEs washed twice with a diethyl-ether/DCM mixture 4:1. The end-capped PBAEs were then dried under vacuum and appeared viscous fluids at room temperature.

PBAE are known to form micelles when in aqueous fluids^33,35^, we hypothesized that the PBAEs-TBO complexes would be constituted by micelles containing TBO with PBAEs on the outer shell.

### Antimicrobial protocol

The organisms used in this study were representatives of pathogens encountered in SSTI. *Escherichia coli* (NCTC10418), *Staphylococcus epidermidis* (ATCC12228), *Pseudomonas aeruginosa* (NCIMB10548), Methicillin Resistant *Staphylococcus aureus* (NCTC12493), *Streptococcus pyogenes* (ATCC19615) and *Acinetobacter baumannii* (NCIMB9214) were stored at − 80 °C. These organisms were subcultured, when needed, on Brain Heart Infusion (BHI) Agar overnight aerobically at 37 °C. The plates were then stored at 4 °C for no more than two weeks. Bacterial suspensions used for the experiments were grown in Brain Heart Infusion (BHI) broth after inoculation with a loopful of cells from a single colony on a BHI plate and incubated aerobically for 24 h at 37 °C statically. These overnight cultures were then diluted 1 in 100 in PBS. The resulting bacterial suspension contained approximately 10^6^ CFU/mL. Each cell suspension was used to disperse pure TBO to concentrations of 0.2 mg/mL or PBAEs-TBO derivatives to an equivalent TBO concentration.

200 μL aliquots of the resulting suspension were immediately poured in a GREINER 96 U-BOTTOM well plate. The well plate was then irradiated with a laser light (633 nm) using a 500 mW red laser (RRL-635 nm-500 nm-1080060, Changchun New Industries Optoelectronics, China) for different time periods (between 10 s to 3 min).

After exposure the bacterial cells (L+S+) were counted through serial dilutions and plating on BHI Agar. Along this test, different experiments were performed including the testing of dark toxicity (labelled with L−S+), samples exposed uniquely to laser light (L+S−) or samples not exposed to either laser light or PBAE (L−S−).

Through the experiments, all prepared samples, well plates and inoculated plates were immediately covered with aluminium foil to reduce biased measuring results due to exposure of any light besides the laser. All plates were incubated after the experiment for 24 h at 37 °C prior to colony forming units counting. All experiments were performed on three independent cultures.

### Spectroscopy

UV–Vis spectrum was determined using the Cary 60 UV–Vis Spectrophotometer (Agilent Technologies, United States).

Absorption of pure TBO and produced TBO–polymers complexes were measured using standard cuvettes. All samples were diluted in PBS to concentrations of equivalent to 20 μg/mL of pure TBO.

### Reactive singlet oxygen species

The generation of reactive oxygen species (ROS) was assessed using a singlet oxygen Sensor Green reagent (SOSG) (Molecular Probes, Unites States). The SOSG was stored at ≤ − 20 °C and protected from light prior to use. The working solution was prepared with methanol to a final concentration of 5 mM. The prepared reagent was further diluted in methanol (1:100) before each experiment. Each sample was dispersed in PBS to a concentration equivalent to 0.2 mg/mL of pure TBO. 100 μL of diluted sample, 100 μL PBS and 20 μL reagent were immediately poured in two adjoining wells of a GREINER 96 U-BOTTOM well plate. The irradiation of one well (L+) was in each case carried out for a period of 1 min with a 500 mW red laser (RRL-635 nm-500 nm-1080060, Changchun New Industries Optoelectronics, China); whilst the other well was covered with aluminium foil (L−). After exposure, ROS were determined using the FLUOstar OPTIMA (BMG Lab technologies, Germany).

### TBO uptake in bacterial cells

10 mL of fresh sterile BHI broth were inoculated with a loopful of cells from a single colony on a BHI plate and incubated aerobically for 24 h at 37 °C statically. The bacterial suspension was then centrifuged with an Avanti J-20XP Centrifuge (Beckmann and Coulter, United States) for duration of 3 min at 2938*g*, afterwards the supernatant was disposed. After one wash and centrifuge with PBS, 1 mL of sample (equivalent concentration of pure TBO of 0.2 mg/mL) was added to the precipitated cells. The resulting solution was vortexed and centrifuged after 3 min exposure. The exposure of samples to cells was limited to 3 min to ensure comparability to other experiments. After two more wash- and centrifugation cycles with PBS, the cells were dissolved in 1 mL 0.1 M NaOH/1% sodium dodecyl sulphate (SDS) and incubated at 37 °C for 24 h to lysate the cells.

The optical density of samples was measured at a 650 nm using a plate reader (Labtech LT5000MS) against a calibration curve prepared using the corresponding bacterial lysate.

### Data analysis

Hieratical clustering (using Manhattan distance between PBAEs and complete distance between clusters) was performed R (ver 4.0) and the “stats” package^36^. Partial Least Square (PLS) regression analysis between PBAEs characteristics and ROS production ratio between PBAE-TBO complexes and pure TBO was carried out using R (ver 4.0)^36^ and the “plsdepot” package^37^.

The physical and chemical parameters of each PBAE constituents (amines and acrylates) were obtained from PubChem library (logP, TPSA, Complexity, Heavy Atom Count, Volume 3D, X_Steric Quadrupole 3D, Y_Steric Quadrupole 3D, Z_Steric Quadrupole 3D) while the parameters related to the basic repeated polymeric unit (amine + acrylate) were calculated through ChemDraw. These included molecular weight (MW), boiling point (BP); melting point (MP); critical volume (CV), Gibb's free energy (GFE), logP, solubility (logS), pKA, molar refractivity (CMR), heat of formation (HtF), TPSA and the ratio between the MW of amine and acrylate. TPSA (topological polar surface area) represents the total area of all polar atoms (mainly oxygen and nitrogen) including their affixed hydrogen atoms while logP is the partition-coefficient between two immiscible phases at equilibrium which is proportional to hydrophobicity of the compound of interest^34^. Further experimentally determined properties of the PBAEs (Mw, Mn and zeta potential) were also added to the properties of PBAE included in the PLS regression.

The minimum number of PLS components to describe the role of different PBAEs chemical–physical properties on the ROS production was determined as the minimum number of PLS components that return at least 80% of the initial input data and an inflection in the scree plot representing the root mean squared error (RMSE) of the predictions vs. number of PLS components was^38,39^.

### In vitro safety assessment

#### Cells culture

Human keratinocytes cells (ATCC PCS-200-011) were grown in Dermal Cell Basal Medium supplemented with Keratinocyte Growth Kit (ATCC); whilst human dermal fibroblast cells were kindly supplied by Prof. Stephens^40^ from Cardiff University and grown in Minimum Essential Medium Eagle (MEM) supplemented with 10% heat-inactivated foetal bovine serum (FBS). Both media were supplemented with 1% penicillin–streptomycin (PS). Both cells lines were incubated at 37 °C in a humidified air atmosphere with 5% CO_2_; media were changed twice per week. 96 well plates were seed with 150 μL containing 10,000 viable cells as determined through trypan blue staining and incubated for 5 days at 37 °C in a humidified air atmosphere with 5% CO_2_; media were changed after 2 days.

#### PDT exposure

Cells were washed three times with PBS and incubated with 100 μL of either TBO 0.2 mg/mL or TBO–PBAE containing TBO 0.2 mg/mL After 2 h, wells were washed once with 100 µL of treatment media and maintained in this media thereafter. The well plate was then irradiated with a laser light (633 nm) using a 500 mW red laser (RRL-635 nm-500 nm-1080060, Changchun New Industries Optoelectronics, China) for 3 min.

Control experiments included testing of dark toxicity (labelled with L−S+), samples not exposed to TBO and uniquely to laser light (L+S−) or samples not exposed to either laser light or PBAE (L−S−).

#### Cytotoxicity assay

20 μL of MTT stock solution (5 mg/mL) were added to each well and incubated in a humidified atmosphere at 37 °C with 5% CO_2_. After 4 h, the supernatant was then removed and 100 μL of dimethyl sulfoxide were added. The absorbance was measured by a spectrophotometer (Lab Tech LT5000MS) at 560 nm; mitochondrial activity was normalised against cell exposed to fresh medium only.

LDH was quantified in the media (LDH released) and after adding the cell lysis solution (LDH total) according to manufacturer’s protocols. Total and released LDH (indicated as LDH_total_ and LDH_released_ respectively) were determined as OD, at 490 nm, after correcting for the reading from the negative control. Cell viability was calculated according to the following equation:^41^

1 $$ viability\; (\% ) = \frac{{LDH_{total} - LDH_{released} }}{{LDH_{total} }} \times 100 $$

### Statistical analysis

Differences among the different samples (L+S+, L−S+, L+S−, L−S−) in the MTT and LDH assays were tested through ANOVA with a significant level p = 0.05.

Multivariate analysis of variance (MANOVA) was carried out to test impact of the different end-capping agents on the antimicrobial activity, TBO cell up-take and ROS production of PBAE-TBO against pure TBO, using the Pillai test with a significant level *p* = 0.05.

## Results

### UV spectra

**Figure 4:**
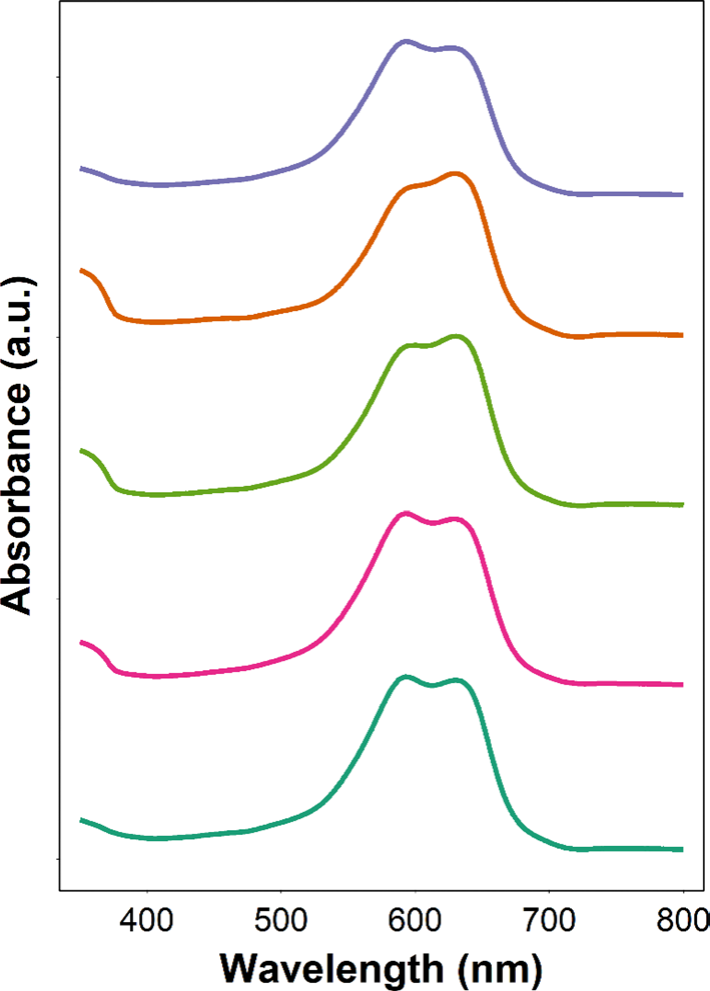
Example of UV spectra of TBO (dark green line) and PBAE-TBO (A3, orange line; B3, purple line; C3, pink line; and A5, light green line).

UV–vis spectra of pure TBO showed a maximum of absorbance at about 633 nm; spectra profiles did not vary when TBO was complexed with PBAE (Fig. 4).

### Antimicrobial activity

**Figure 5:**
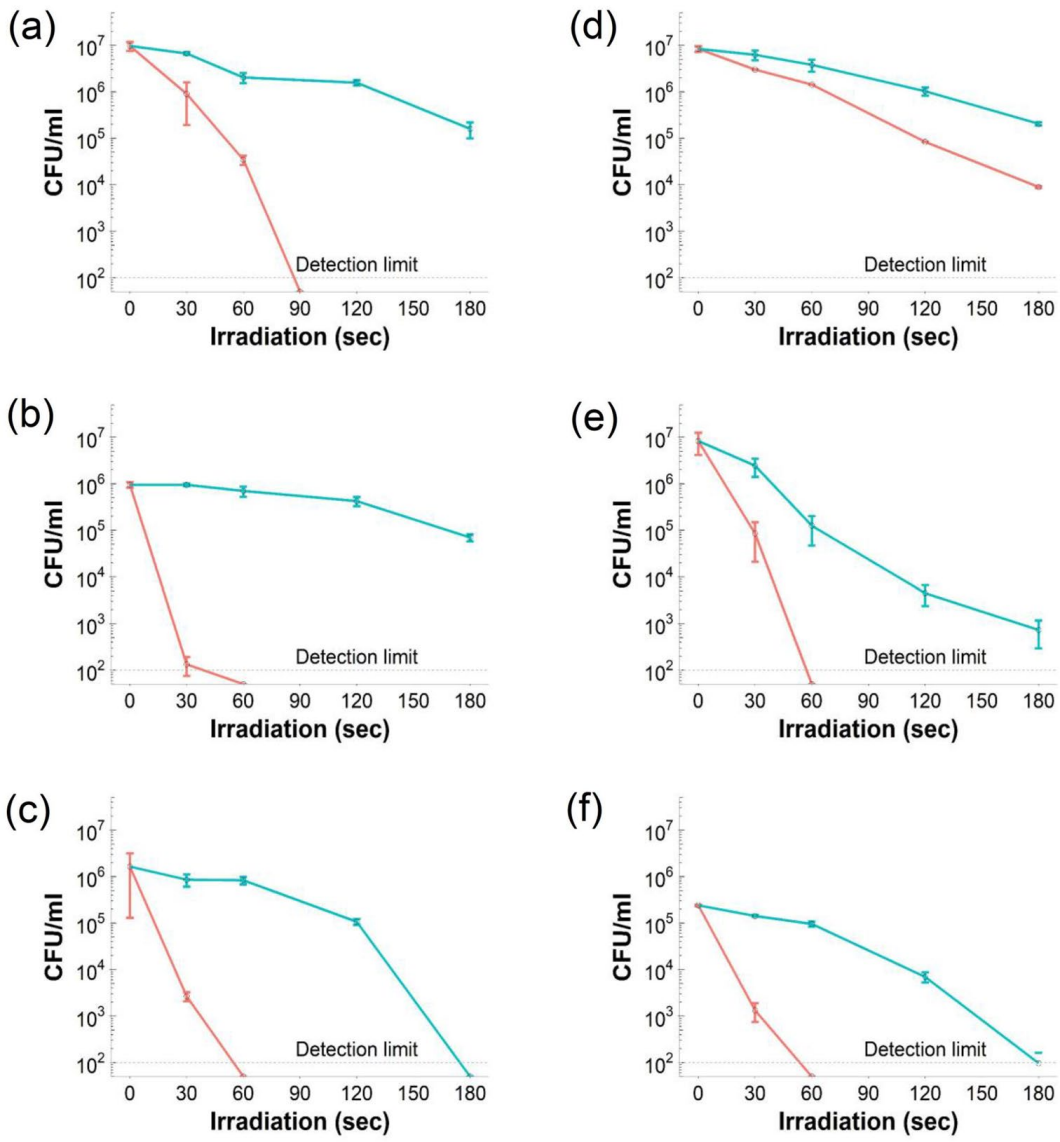
Antimicrobial activity of TBO (blue circles) and A3-e1-TBO (orange circles) against (**a**) *E. coli,* (**b**) *P. aeruginosa*, (**c**) *A. baumannii*, (**d**) *MRSA*, (**e**) *S. epidermidis* and (**f**) *S. pyogenes* (mean ± SD, n = 3).

In all six species tested (Fig. 5), viable bacteria decreased with the increase of irradiation time when in the presence of TBO (L+S+); inactivation rates due to irradiation and TBO were higher for three Gram+ pathogens employed (*S. epidermidis*, MRSA and *S. pyogenes*) than the Gram- (*P. aeruginosa*, *E. coli* and *A. baumannii*).

In all cases, samples exposed to the polymer-TBO complex or to pure TBO without irradiation (L−) did not return any cell reduction over the time considered. In comparison, irradiated (L+) samples show tendencies of progressive reduction. The comparison with pure TBO showed an obvious improvement in the antimicrobial effect of laser exposure in all cases when the same amount of photosensitiser was employed along with A3-e1. Furthermore, when cells were exposed to laser light without the presence of photosensitiser (L+S−) did not reveal any antimicrobial activity. Furthermore, PBAE alone (without TBO) did not exhibit antimicrobial activity towards any of the pathogens tested either in the dark or after laser irradiation.

### PBAE optimisation

**Figure 6:**
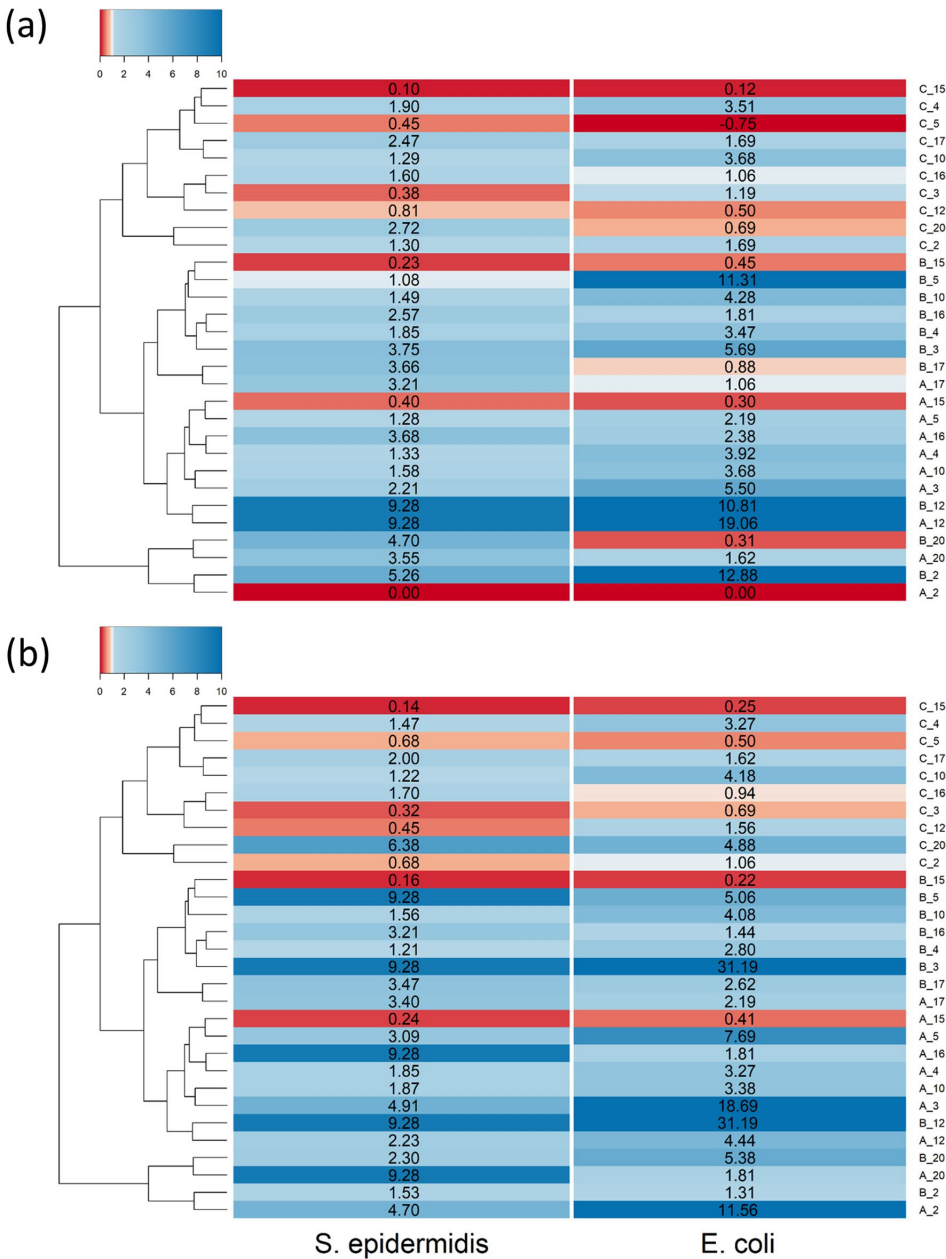
Log_10_ reduction of viable cells after 30 s laser exposure of *E. coli* and *S. epidermidis* comparted to pure TBO when exposed to different TBO–PBAEs end-capped with (**a**) e1 and (**b**) e2.

The comparison of the efficacy of other PBAE as delivery system of TBO for antimicrobial PDT against an example of Gram− (*E. coli*) and an example of Gram+ (*S. epidermidis*) is shown in Fig. 6. Clustering of the PBAE synthesised revealed that main difference among polymers was the acrylate component with Poly(ethylene glycol) diacrylate (acrylate C) forming a cluster more distant than 1,6-Hexanediol diacrylate (acrylate B) or 1,4-Butanediol diacrylate (acrylate A); the couples of amines 4,4′-Trimethylenedipiperidine (amine 2) and *N*,*N*-bis[(3-methylamino)propyl]methylamine (amine 20) along with 3-(dimethylamino)-1-propilamine (amine 5) and cyclopentylamine (amine 15) resulted in similar PBAE than any of the other amines.

Using similar amine and end-capping agent for the preparation of PBAE, 1,6-Hexanediol diacrylate (acrylate B) was usually more effective than 1,4-Butanediol diacrylate (acrylate A) with Poly(ethylene glycol) diacrylate (acrylate C) returning the worst antimicrobial activity of the three. Most of the PBAE-TBO complexes improve the antimicrobial activity compared to the same amount of pure TBO. PBAEs end-capped with e2 had better antimicrobial activity that the respective counter-part end-capped with e1 (*p* < 0.05).

### Cell uptake

**Figure 7:**
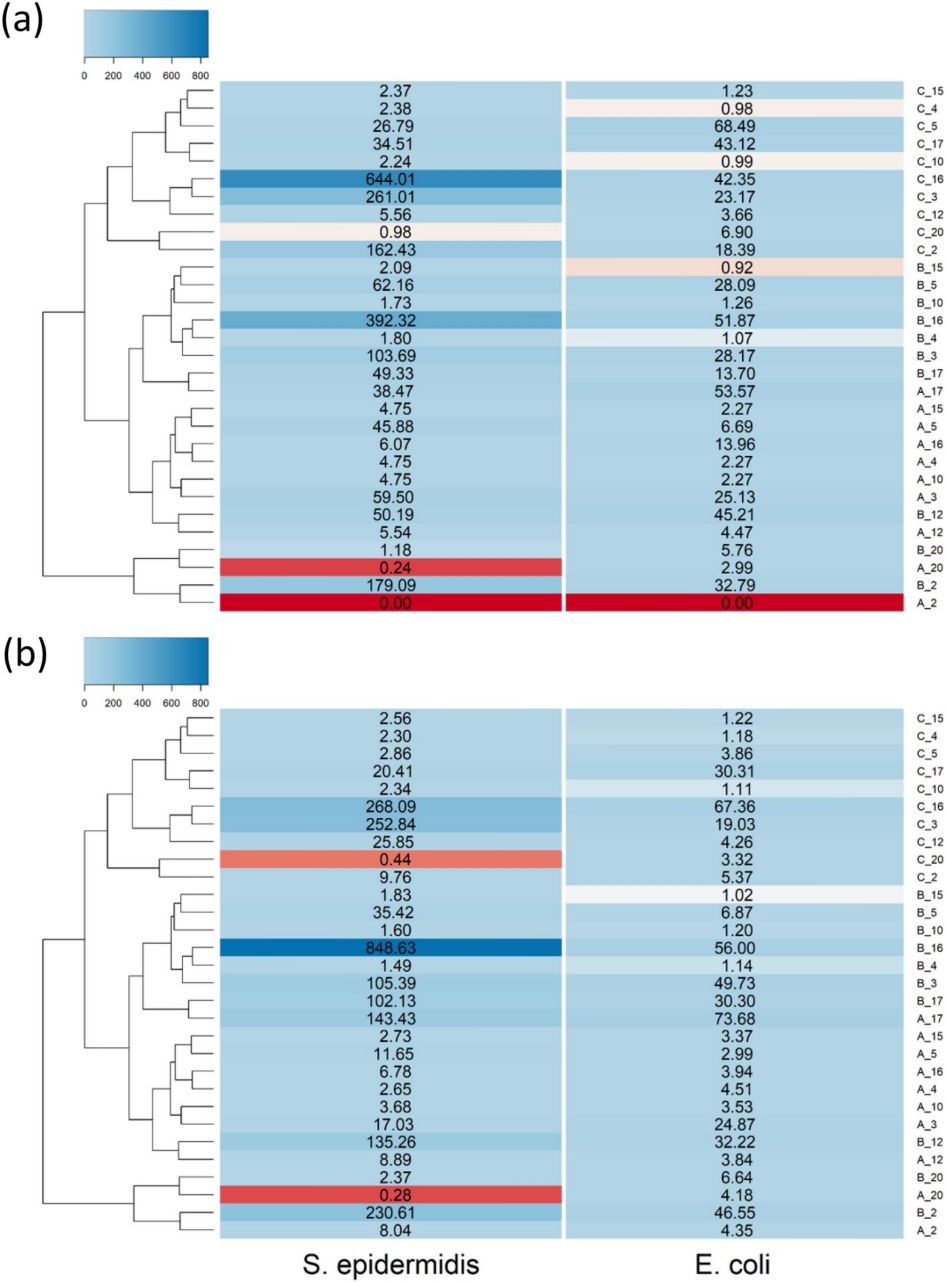
Uptake of TBO for *E. coli* and *S. epidermidis* comparted to pure TBO when exposed to different TBO–PBAEs end-capped with (**a**) e1 and (**b**) e2.

All PBAE tested increased the uptake of TBO in both *E. coli* and *S. epidermidis* (Fig. 7), polymer exhibiting 1,6-Hexanediol diacrylate in their backbone performed better than those with 1,4-Butanediol diacrylate in most of the cases and the adjuvant effect of PBAE is remarkably greater in *S. epidermidis* than in *E. coli.* Poly(ethylene glycol) diacrylate generally did not enhance TBO cell uptake. PBAEs end-capped with e2 resulted in higher TBO cellular uptake that the respective counter-part end-capped with e1 (*p* < 0.05).

### Reactive oxygen species production

**Figure 8:**
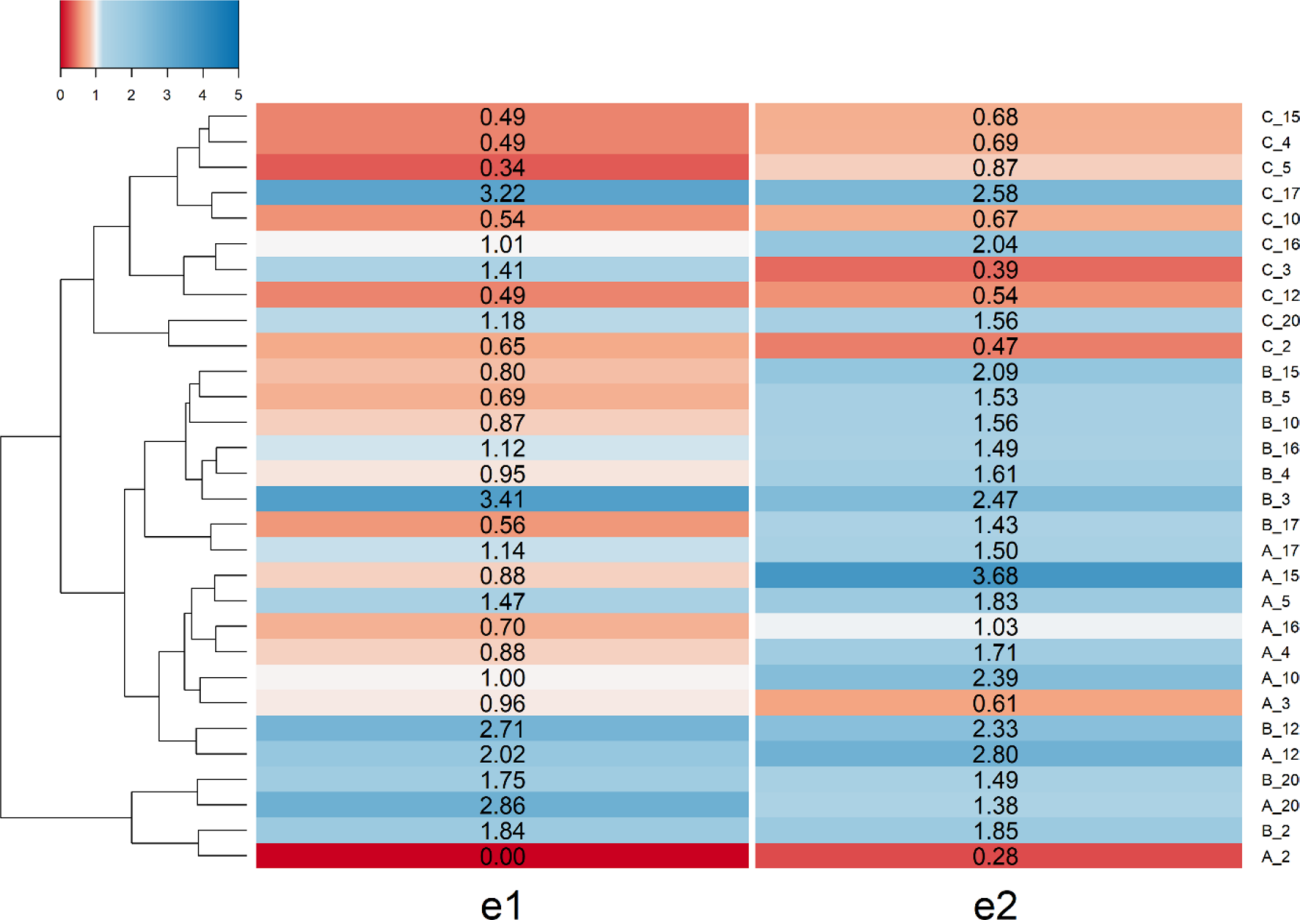
ROS produced by different PBAEs-TBO after 1 min irradiation compared to pure TBO.

The amount of ROS generated by pure TBO only PBAE-TBO complexes is shown in Fig. 8 and reveals a strong dependence on the presence and structure of the PBAE. In most of the cases, TBO complexes with polymers prepared with 1,6-Hexanediol diacrylate performed better than those prepared with 1,4-Butanediol diacrylate. TBO in combination with complexes of PBAEs end-capped with e2 resulted in higher ROS that the respective counter-part end-capped with e1 (*p* < 0.05).

No ROS were detected when only PBAE were exposed to laser light, hence PBAE are not photosensitisers. This demonstrate that PBAE do not only act in enhancing TBO uptake by cells but also have a direct role in the mechanism of radicals formation by PDT.

**Figure 9:**
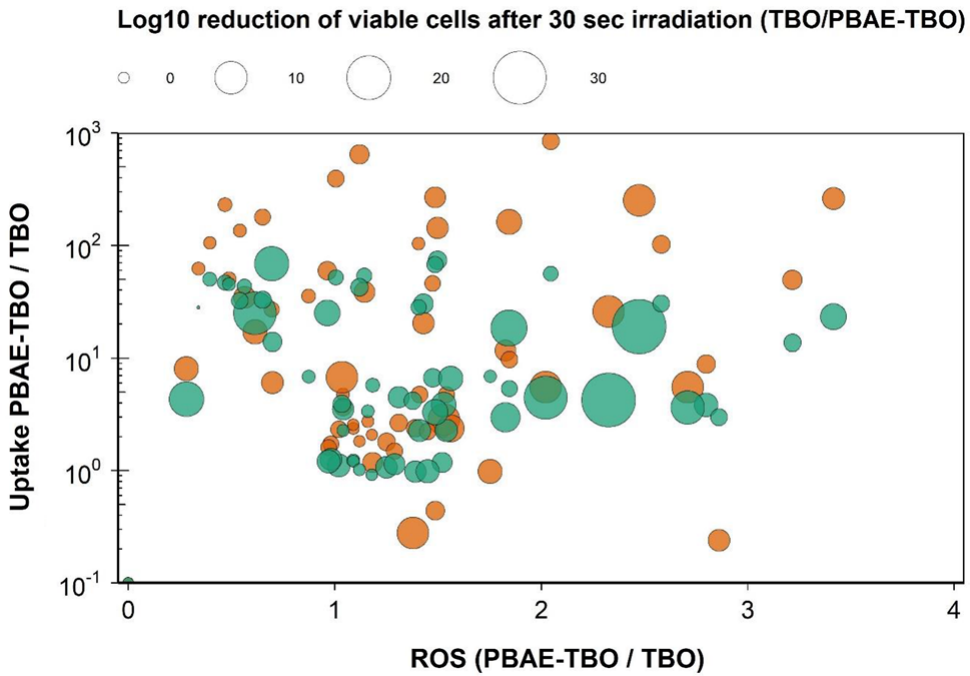
Correlation of ROS produced and TBO uptake on antimicrobial activity (ratio of log10 reduction after 30 s laser irradiation in the presence of TBO or PBAE-TBO) of *E. coli* (green circles) and *S. epidermidis* (orange circles) by different PBAEs-TBO.

Antimicrobial activity of PBAE-TBO complexes on either *S. epidermidis* or *E. coli* was not solely depended on TBO uptake but was a combination of both uptake and ROS as not linear correlation was noticeable (Fig. 9).

### PBAEs feature selection in ROS production

PLS model predictions of ROS production against the experimental data improved with increasing number of PLS components as represented by the increasing MRSE and R^2^ (Table S2 and Supplementary material). The scree plots exhibited an inflection at transition from 11 to 12 PLS components (Supplementary material); therefore 11 PLS components were chosen to describe the variation within this dataset of PBAE-TBO complexes. The experimental data of ROS production by PBAE-TBO synthesised were closely match by the predictions (Supplementary material); the residual did not reveal clear patterns (Supplementary material) and followed a normal distribution (Supplementary material).

**Figure 10:**
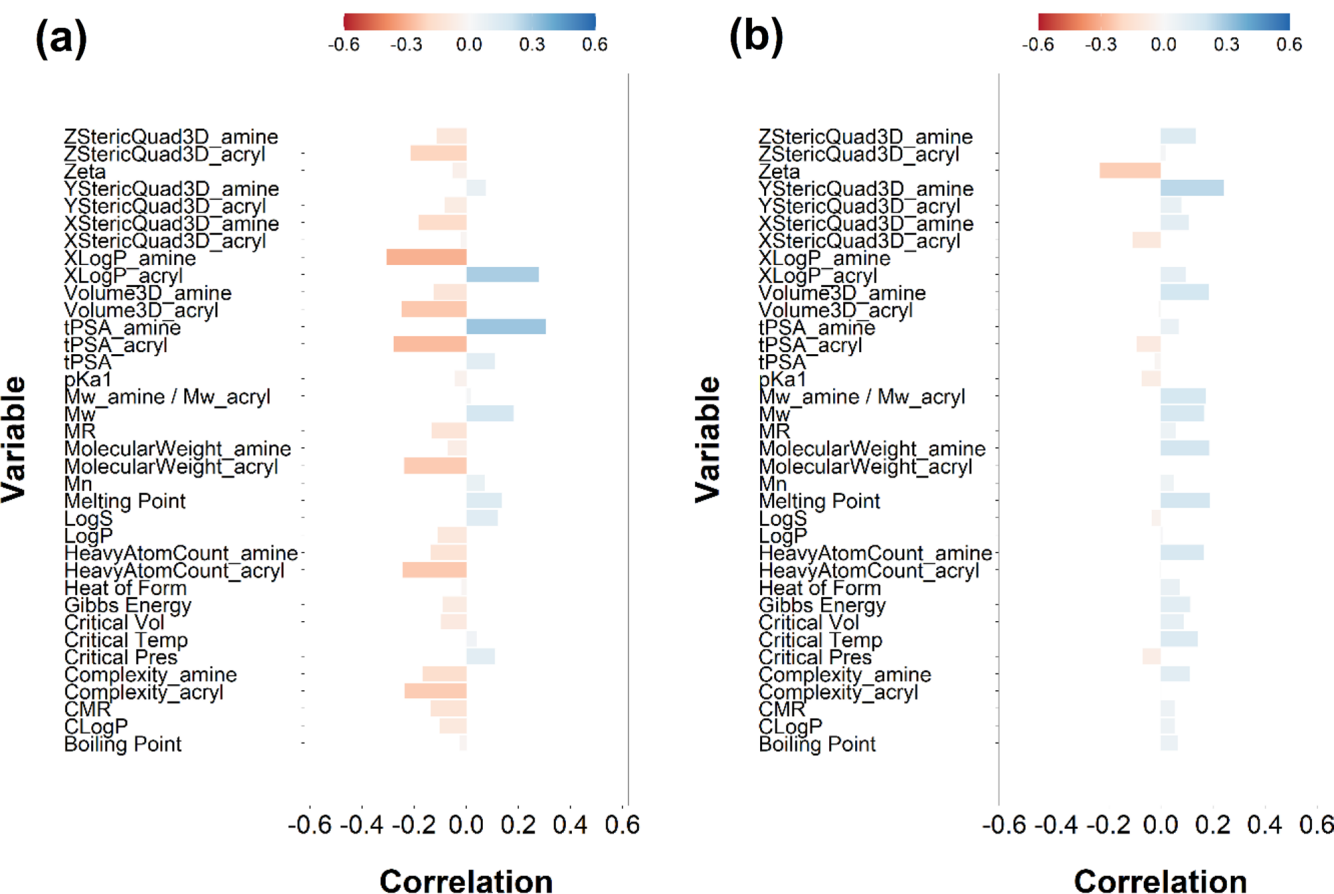
PLS derived correlations for the different PBAEs characteristic (input parameters within the model) and ROS production for PBAE end-capped with e1 (**a**) and e2 (**b**).

For both end-capping agents, PBAE zeta potential was negatively correlated to the ROS production of the TBO–PBAE complexes after laser irradiation, on the contrary LogP and steric occupation (StericQuad3D) of the amine component of the polymer was positively correlated to ROS formation (Fig. 10).

### In vitro test on skin cells

**Figure 11:**
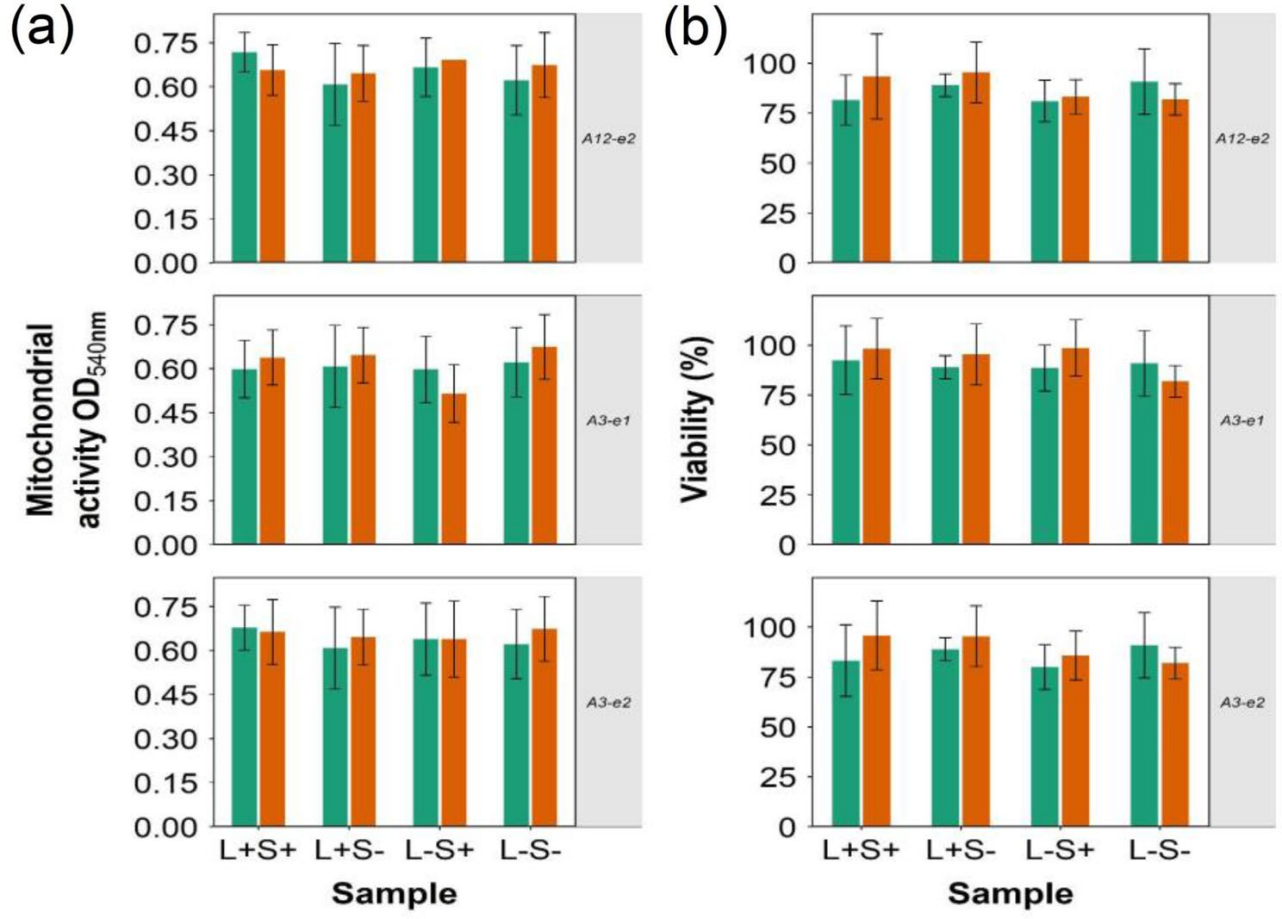
MTT and LDH on keratinocytes (green circles) and fibroblasts (orange circles) exposed to TBO and TBO–PBAEs (mean ± SD, n = 3).

Mitochondrial activity, estimated through MTT assay, and cell viability determined through LDH assay of both keratinocytes and fibroblasts were not impacted (p > 0.05) by exposure to a selection of the TBO–PBAE prepared, additionally not dark toxicity was detected in either cell lines (Fig. 11).

## Discussion

aPDT has numerous benefits as potential alternative to antibiotics for the treatment of infections such as skin and tissues that can be accessed with light sources^10,12,42^; however a critical drawback is the still relative long treatment time required (in the order of tens of minutes) that impacts the largescale application of this technology^26^. The design of novel and more efficient photosensitisers is generally accompanied by elevated drug costs; similarly, the use of drug delivery systems brings additional costs that must be offset by the enhanced efficacy. We have brought a new approach to boost aPDT efficacy through the use of polymers enhancing intracellular drug uptake; our starting point was the use of a photosensitiser commercially available and approved for use in humans, specifically TBO (widely used, inexpensive and with a very well-known safety profile)^43^, and polymers proven biocompatible and low cost such as PBAE. The safety profile of the compounds used in this technology offers both a shorter route to market and a high likelihood of future in vivo trials of this approach to prove safety while the use of low-cost chemicals also improves opportunities to be cost-effective.

We firstly validated the hypothesis of using PBAE to augment PDT on a variety of pathogens representing those most commonly encountered in SSTI^44,45^; subsequently the role of polymer backbone constituents was assessed through a broad library of PBAE and a new discovery, the impact on ROS, beside TBO uptake was also observed. PBAE features critical in this newly noticed phenomenon were assessed through PLS, one of chemometrics data analytic techniques that have been employed in drug design and analytical chemistry^34,46,47^, to also elicit possible mechanism. Initial in-vitro tests on keratinocytes cells and fibroblasts were also carried out exposing the cells to a selection of the PBAE-TBO complexes that exhibited the greatest enhancement of antimicrobial activity; in order to gather more robust safety data, two independent assays (MTT and LDH) were utilised with both showing the safety of the complexes PBAE-TBO on skin tissue cells.

The higher vulnerability of Gram+ over Gram− observed in this study (Fig. 5) was expected in line with several studies^48–50^ and is widely considered to be explained by the relatively porous layer of peptidoglycan and lipoteichoic acid constituting the cell wall of Gram+ that allows the photosensitiser to cross the cell wall compared to the cell envelope of Gram- bacteria that exhibit higher resistance to photosensitiser uptake^48^.

PBAE were first described as gene transfer vectors^32^; subsequent evidence has validated such properties and identified key features and optimised the polymer chain backbone in order to maximise the opportunities offered by such polymers to transfer genes into target cells^35,51^. Further applications of PBAEs have been proposed for cancer drug delivery^52–55^, in layer-by-layer deposition of drug releasing coatings^41,56–61^ and drug localisation in cartilage^33,34^; currently no application of these polymers in antimicrobial technologies has been reported. Depending on the application, different PBAE features have been shown to pivot the outcome; for example PBAEs belonging to the family of B3 and A16 are known cell uptake enhancer in DNA delivery^32,35,51^ while A5 and B5 are better at driving drug uptake into cartilage^34^; additionally it has also been proven that end-capping has a further critical role^34,35^. Because of the essential role on TBO uptake into bacterial cells in controlling aPDT effect, it was not unexpected that polymer synthesised with acrylates such as A and B with amines such as 3 and 16 had the greatest impact on TBO uptake (Fig. 7).

All three constituents of PBAEs, namely diacrylate, amine and end-capping, influence all the outcomes investigated here (Figs. 6, 7, 8) in agreement with previous results on the various applications of PBAEs^34,35^. None of the tested PBAEs have antimicrobial activity when used alone; furthermore, the overall performance of PBAE-TBO in bacteria inactivation is the combination of two phenomena in which PBAEs are responsible (Fig. 9), one is the cell uptake and another, seen for the first time in this work, in the generation of ROS by an irradiated photosensitisers. Because of the high reactivity of the singlet state, most of the energy absorbed by PS is re-emitted without resulting in any ROS. As PBAEs do not exhibit photosensitiser properties as shown by the absence of ROS for pure PBAE irradiated and the UV spectra of TBO is unaffected by the complexation with PBAE (Fig. 4), in order to explain the increase ROS formation, we hypothesised that PBAE may impact the stability of the TBO single/triple states resulting in a more efficient conversion of the light energy into ROS instead of re-emission; this charge separation and recombination phenomenon would be similar to those observed in polymers capped with light absorbing moieties^62^. Alternatively, PBAE may also interact in the mechanisms PS releases the absorbed energy from the light resulting in the formation of PBAE radicals with longer half-life that react with the bacterial cells wall causing the microorganism inactivation. The results of the PLS indicating that PBAE zeta potential is inversely correlated to the ROS production suggests that the electrostatic attraction between PBAE and TBO (positively charged) is necessary for the augmented ROS formation by TBO when in presence of PBAEs. Moreover, the high correlation between TSPA (the level of polar surface) characteristics of PBAE components further support this hypothesis; under this hypothesis, it was expected that the correlation between TPSA of acrylates and amine had opposite signs as amine have positive charges whilst acrylates only exhibit oxygen atoms that results in negative charges.

PBAEs are biodegradable, therefore, it is expected that storage of aqueous suspensions of PBAE-TBO would exhibit progressively diminishing improvements on aPDT. This obstacle could easily be overcome as we envisage the storage of the prepared PBAE-TBO dried in airtight containers with PBS added just before use.

The duration of the exposure time necessary to reach satisfactory antimicrobial effects is key in controlling the number of patients that can be treated given the available beds/treatment bays capacity; thus, increasing inactivation rates and, consequently, shortening treatment times, reduces the indirect costs of the treatment or the required investments to set-up facilities with a set turn-over of patients. Most of the technologies developed to enhance photosensitiser uptake rely on the conjugation of the light activated compound to a bacterial targeting polymer^63^, antimicrobial polypeptides^64–66^ or a bacterial wall disruption agent (i.e. gentamicin^67^ or vancomycin^68–70^). Another type of approach is the encapsulation of the photosensitiser in liposomes or micelle that drive the light activate compound inside the bacterial cell^28,71,72^. Compared to these approaches, the simple use of PBAE complexation with TBO that we developed removes the need of the conjugation or the encapsulation step thus reducing costs and maximising yield.

## Conclusions

Antimicrobial PDT has the potential to replace antibiotics in the treatment of skin and tissue infections helping the fight against antibiotic resistance; current applications are still significantly restricted by long exposure time necessary.

We developed a new approach to improving aPDT efficacy thought the use of PBAE to improve bacterial uptake of photosensitisers resulting in shorter exposure times that, in turn, men higher treatment bays turnover. Reducing treatment times means that more patients can be treated with the currently available resources maximising their return. Further benefits of this technology over the current treatments of chronic wounds including the use of Iodine (Povidone), KMnO_4_, bleach or silver are the safety, low cost and biological tolerance (linked to the biological compatibility of the degradation products). Additionally, our technology has the advantage that the PBAE-TBO is already a gel and such its topical application is very easy and does not require further formulation into a gel.

## Supplementary Information

Supplementary Information.
