# Supplementary material for: Amplify antimicrobial photo dynamic therapy efficacy with poly-beta-amino esters (PBAEs)
Source: Sci Rep. 2021 Mar 31;11:7275. doi: 10.1038/s41598-021-86773-3 (PMC8012660; doi:10.1038/s41598-021-86773-3)
Supplement: Supplementary file 1 — Supplementary Information. [file 41598_2021_86773_MOESM1_ESM.docx]

**Amplify antimicrobial Photo Dynamic Therapy efficacy with Poly-beta-amino esters (PBAEs)**

by

Stefano Perni^1^, Emily C. Preedy^1^, Polina Prokopovich^1,*^

^1^ Cardiff School of Pharmacy and Pharmaceutical Sciences, Cardiff University, UK

# Supplementary information

| **PBAE** | **Zeta potential (mV)** | **Mn** | **Mw** | **PDI** |
| --- | --- | --- | --- | --- |
| A2 | 8.94 | 5988 ± 426 | 9156 ± 112 | 1.54 ± 0.12 |
| A3 | 12.77 | 5499 ± 313 | 8389 ± 346 | 1.53 ± 0.09 |
| A4 | 14.37 | 5710 ± 266 | 10643 ± 684 | 1.87 ± 0.15 |
| A5 | 16.90 | 4941 ± 274 | 7619 ± 170 | 1.55 ± 0.10 |
| A10 | 6.27 | 3820 ± 158 | 6831 ± 334 | 1.79 ± 0.08 |
| A12 | 4.09 | 6149 ± 484 | 8077 ± 433 | 1.32 ± 0.13 |
| A15 | 13.97 | 5273 ± 277 | 8026 ± 141 | 1.53 ± 0.07 |
| A16 | 9.29 | 6168 ± 413 | 7767 ± 547 | 1.27 ± 0.17 |
| A17 | 9.55 | 3370 ± 165 | 8466 ± 576 | 2.52 ± 0.20 |
| A20 | 5.93 | 6380 ± 30 | 10426 ± 464 | 1.63 ± 0.08 |
| B2 | 10.67 | 9004 ± 674 | 11459 ± 727 | 1.28 ± 0.17 |
| B3 | 8.87 | 6728 ± 301 | 9043 ± 248 | 1.35 ± 0.09 |
| B4 | 14.60 | 6480 ± 542 | 7181 ± 505 | 1.12 ± 0.14 |
| B5 | 20.10 | 7351 ± 371 | 12196 ± 610 | 1.66 ± 0.10 |
| B10 | 11.47 | 2305 ± 149 | 2699 ± 191 | 1.17 ± 0.03 |
| B12 | 10.60 | 4415 ± 172 | 6786 ± 350 | 1.54 ± 0.11 |
| B15 | 15.70 | 1989 ± 77 | 1913 ± 57 | 1.01 ± 0.07 |
| B16 | 12.87 | 3543 ± 120 | 5429 ± 54 | 1.53 ± 0.04 |
| B17 | 10.35 | 3499 ± 254 | 6761 ± 383 | 1.94 ± 0.20 |
| B20 | 10.47 | 8678 ± 505 | 11574 ± 295 | 1.34 ± 0.11 |
| C2 | 8.23 | 2190 ± 101 | 3982 ± 35 | 1.82 ± 0.08 |
| C3 | 3.81 | 3739 ± 165 | 6045 ± 320 | 1.62 ± 0.10 |
| C4 | 3.81 | 2353 ± 161 | 2540 ± 161 | 1.09 ± 0.14 |
| C5 | 9.14 | 4178 ± 139 | 6317 ± 469 | 1.51 ± 0.12 |
| C10 | 5.33 | 1818 ± 82 | 2151 ± 157 | 1.18 ± 0.04 |
| C12 | 7.14 | 2951 ± 164 | 4856 ± 253 | 1.65 ± 0.11 |
| C15 | 5.30 | 1354 ± 60 | 1698 ± 84 | 1.25 ± 0.07 |
| C16 | 4.21 | 2401 ± 63 | 4495 ± 191 | 1.87 ± 0.10 |
| C17 | 7.29 | 2542 ± 33 | 3140 ± 200 | 1.23 ± 0.07 |
| C20 | 6.57 | 5381 ± 210 | 9312 ± 683 | 1.74 ± 0.19 |

Table S 1. Weight average molecular weight (Mw), number average molecular weight (Mn) and polydispersity index (PDI) of the PBAEs synthesised. (mean ± SD, n = 3)

#
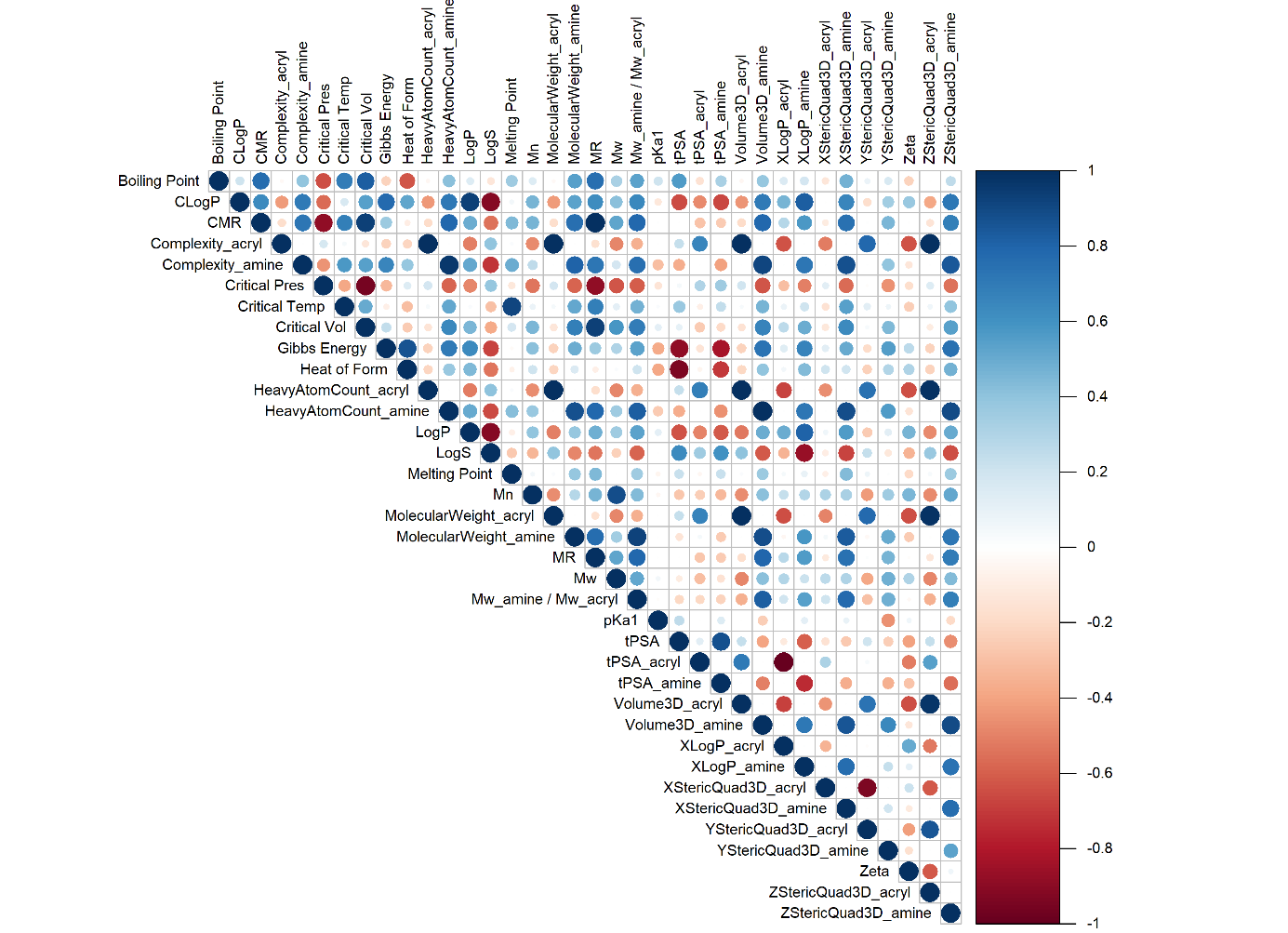


Figure S 1. Correlation plot of the PBAEs properties.

Table S 2. R^2^ on ROS production and variables associated to each PLS components.

| Number of components | X-R^2^ | X- R^2^ cum | Y-R^2^ | Y- R^2^ cum |
| --- | --- | --- | --- | --- |
| 1 | 0.21 | 0.21 | 0.14 | 0.14 |
| 2 | 0.27 | 0.48 | 0.06 | 0.20 |
| 3 | 0.16 | 0.65 | 0.06 | 0.26 |
| 4 | 0.12 | 0.77 | 0.04 | 0.30 |
| 5 | 0.03 | 0.80 | 0.05 | 0.35 |
| 6 | 0.04 | 0.84 | 0.03 | 0.38 |
| 7 | 0.04 | 0.88 | 0.04 | 0.42 |
| 8 | 0.03 | 0.92 | 0.06 | 0.48 |
| 9 | 0.05 | 0.97 | 0.03 | 0.51 |
| 10 | 0.01 | 0.97 | 0.08 | 0.59 |


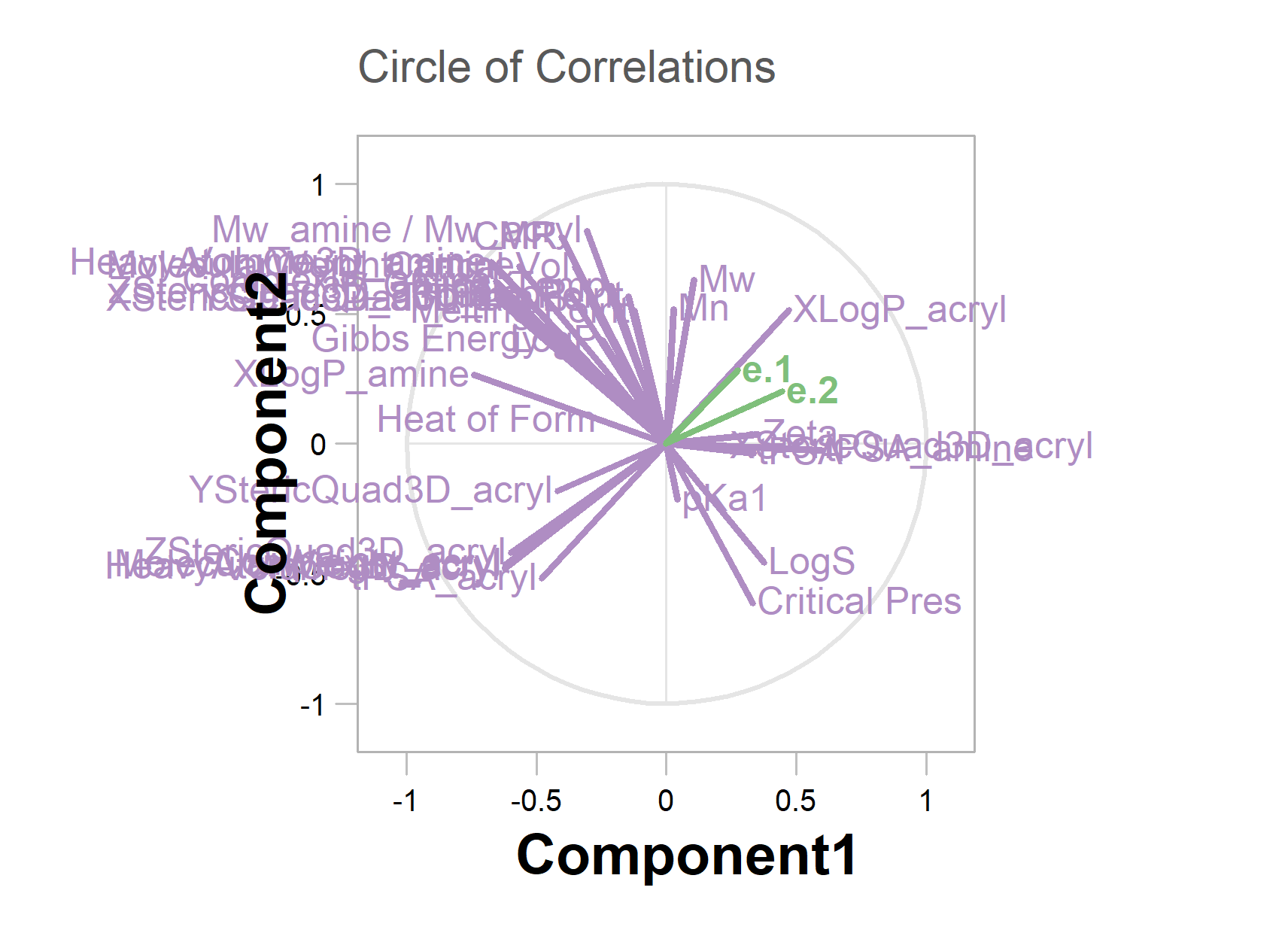


Figure S 2. Loading plot of the first two PLS components describing the relationships between input parameters for the set of PBAEs tested (purple) and the predicted ROS production ratio for the two end-capping agents (green).


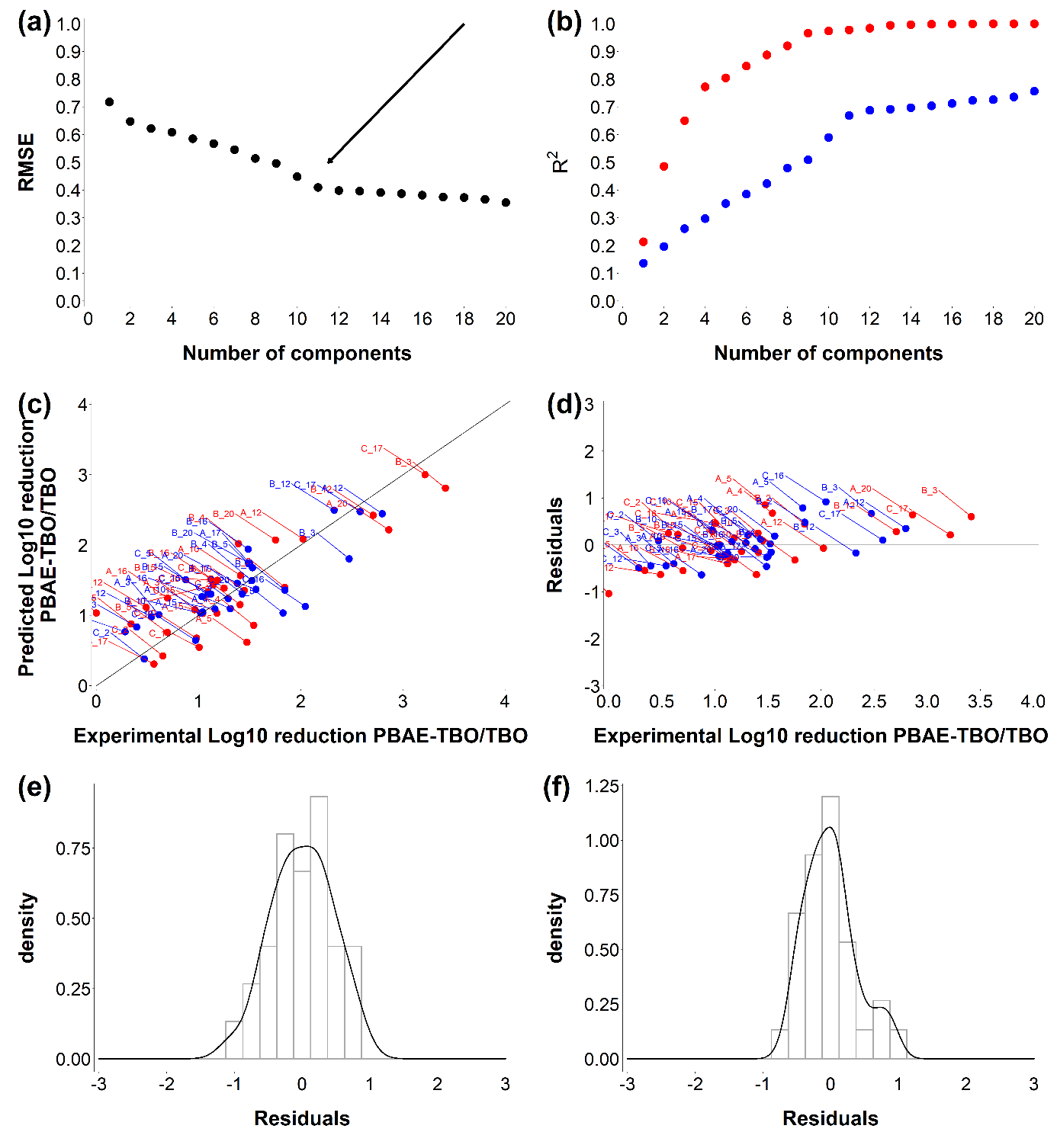


Figure S 3. Scree plot of PLS analysis with respect to number of components in the PLS regression and the RMSE of the prediction; curve inflection (elbow) indicated with an arrow (a); R^2^ of experimental vs PLS predicted ratio of ROS production using different PBEAs end-capped with e1 (red) and e2 (blue) (b); Experimental and PLS predicted ratio of ROS production using different PBEAs end-capped with e1 (red) and e2 (blue) (c); Residuals plot of PLS predicted vs. experimental ratios of ROS production using different PBEAs end-capped with e1 (red) and e2 (blue) (d); Distribution of residuals of PLS predicted ratio of ROS production vs. experimental for PBAE end-capped with e1 (e) and e2 (f).
